# 6-Hydroxy­salvinolone^1^

**DOI:** 10.1107/S1600536809034990

**Published:** 2009-09-09

**Authors:** Abdul Wahab Salae, Suchada Chantrapromma, Hoong-Kun Fun, Chatchanok Karalai

**Affiliations:** a Crystal Materials Research Unit, Department of Chemistry, Faculty of Science, Prince of Songkla University, Hat-Yai, Songkhla 90112, Thailand; b X-ray Crystallography Unit, School of Physics, Universiti Sains Malaysia, 11800 USM, Penang, Malaysia

## Abstract

The title compound {systematic name: 5,6,10-trihydr­oxy-7-iso­propyl-1,1,4a-trimethyl-2,3,4,4a-tetra­hydro­phenanthren-9(1*H*)-one}, C_20_H_26_O_4_, is a diterpenoid which was isolated from the roots of *Premna obtusifolia*. The mol­ecule has three fused six-membered rings; the cyclo­hexane ring is in a twisted-boat conformation and the cyclo­hexene ring adopts a sofa form. Intra­molecular O—H⋯O hydrogen bonds generate two *S*(5) ring motifs. In the crystal, mol­ecules are linked into infinite one-dimensional chains along the [001] direction by O—H⋯O hydrogen bonds and weak C—H⋯O inter­actions.

## Related literature

For hydrogen bond motifs, see: Bernstein *et al.* (1995 ▶). For ring conformations, see: Cremer & Pople (1975 ▶). For bond-length data, see: Allen *et al.* (1987 ▶). For background to diterpenes and their activities, see: Fraga *et al.* (2005 ▶); Hueso-Rodríguez *et al.* (1983 ▶); Topcu & Ulubelen (1996 ▶). For the stability of the temperature controller used in the data collection, see Cosier & Glazer, (1986 ▶).
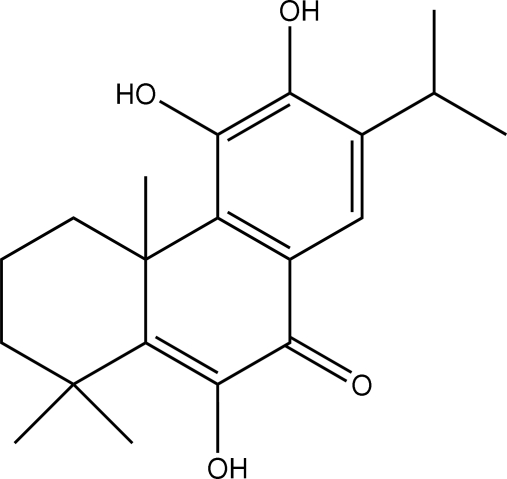


## Experimental

### 

#### Crystal data

C_20_H_26_O_4_
*M* *_r_* = 330.41
Orthorhombic, 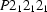

*a* = 9.4946 (1) Å
*b* = 13.1716 (1) Å
*c* = 13.8124 (1) Å
*V* = 1727.37 (3) Å^3^
*Z* = 4
Mo *K*α radiation
μ = 0.09 mm^−1^
*T* = 100 K
0.35 × 0.30 × 0.27 mm

#### Data collection

Bruker APEXII CCD area-detector diffractometer
Absorption correction: multi-scan (*SADABS*; Bruker, 2005 ▶) *T*_min_ = 0.970, *T*_max_ = 0.977
36584 measured reflections
3890 independent reflections
3530 reflections with *I* > 2σ(*I*)
*R*_int_ = 0.031

#### Refinement

*R*[*F* ^2^ > 2σ(*F* ^2^)] = 0.039
*wR*(*F* ^2^) = 0.114
*S* = 1.09
3890 reflections
230 parameters
H atoms treated by a mixture of independent and constrained refinement
Δρ_max_ = 0.36 e Å^−3^
Δρ_min_ = −0.41 e Å^−3^

### 

Data collection: *APEX2* (Bruker, 2005 ▶); cell refinement: *SAINT* (Bruker, 2005 ▶); data reduction: *SAINT*; program(s) used to solve structure: *SHELXTL* (Sheldrick, 2008 ▶); program(s) used to refine structure: *SHELXTL*; molecular graphics: *SHELXTL* software used to prepare material for publication: *SHELXTL* and *PLATON* (Spek, 2009 ▶).

## Supplementary Material

Crystal structure: contains datablocks global, I. DOI: 10.1107/S1600536809034990/sj2639sup1.cif

Structure factors: contains datablocks I. DOI: 10.1107/S1600536809034990/sj2639Isup2.hkl

Additional supplementary materials:  crystallographic information; 3D view; checkCIF report

## Figures and Tables

**Table 1:** Hydrogen-bond geometry (Å, °)

*D*—H⋯*A*	*D*—H	H⋯*A*	*D*⋯*A*	*D*—H⋯*A*
O1—H1*O*1⋯O2	0.86 (2)	1.97 (2)	2.5626 (13)	124.5 (19)
O3—H1*O*3⋯O2^i^	0.82	1.93	2.7008 (13)	156
O4—H1*O*4⋯O3	0.81 (2)	1.96 (2)	2.5677 (13)	131 (2)
C14—H14*A*⋯O3^ii^	0.93	2.57	3.4712 (14)	163
C15—H15*A*⋯O2^i^	0.98	2.45	3.1908 (14)	132
C18—H18*A*⋯O1	0.96	2.24	2.9053 (18)	125
C19—H19*B*⋯O1	0.96	2.53	3.1360 (17)	121
C20—H20*C*⋯O4	0.96	2.44	3.0838 (16)	124

Symmetry codes: (i) 
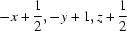
; (ii) 
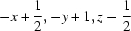
.

**Fig. 1.**
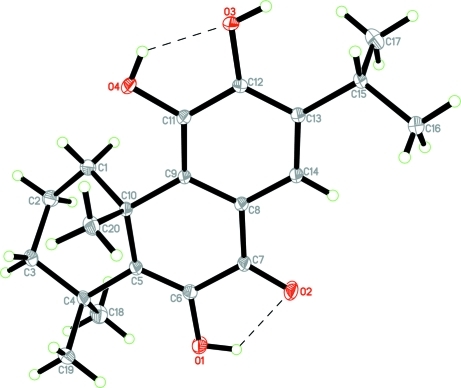
The structure of (I), showing 50% probability displacement ellipsoids and the atom-numbering scheme. Intramolecular hydrogen bonds are shown as dashed lines.

**Fig. 2.**
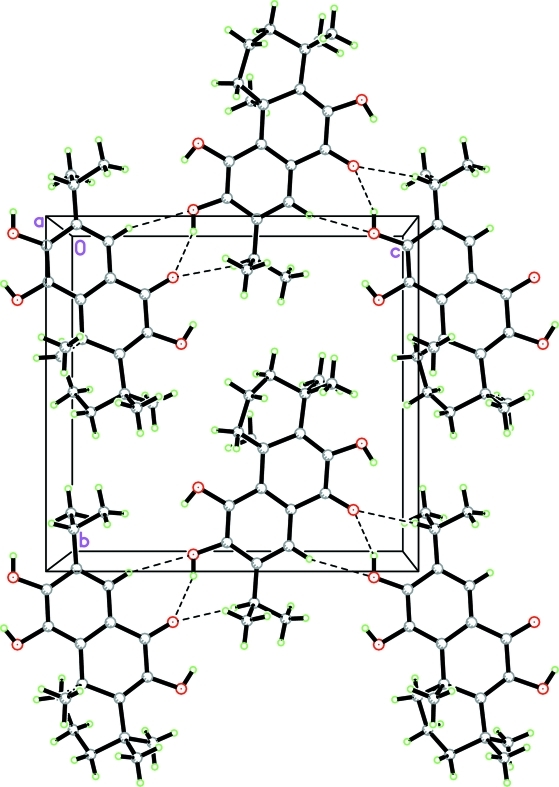
The crystal packing of (I) viewed along the a axis, showing one dimensional chains along the [0 0 1] direction. Hydrogen bonds are shown as dashed lines.

## supplementary crystallographic information

### Comment

During the course of our studies on the chemical constituents and bioactive compounds from Thai medicinal plants, the title diterpenoid compound (I) known as 6-hydroxysalvinolone (Topcu & Ulubelen,  1996) or 14-deoxycoleon U (Fraga *et al.*, 2005; Hueso-Rodríguez *et al.*, 1983), was isolated from the roots of *Premna obtusifolia*. The previous report (Fraga *et al.*, 2005) shows that this compound exhibits insecticidal activity. We report herein the crystal structure of (I).

The molecule of (I) has three fused six membered rings (Fig. 1). The cyclohexane ring is in a twisted boat conformation with the puckering parameters Q = 0.6874 (13) Å, θ = 96.44 (11)° and φ = 278.29 (11)° (Cremer & Pople, 1975) whereas the cyclohexene ring (C5–C10) adopts a sofa form with the slightly puckered C5 atom having the maximum deviation of -0.058 (1) Å. The C5···C14 ring system is nearly planar, rms deviation 0.0216 (13) Å and the O1, O3 and O4 hydroxyl O atoms lie close to this plane with deviations +0.0393 (13) for O1, -0.0316 (11) for O3 and +0.0305 (11) Å for O4. The bond angles around C6 are indicative of *sp*^2^ hybridization for this atom. The orientation of the propanyl group is described by the torsion angles C14–C13–C15–C16 = -25.4 (2) and C14–C13–C15–C17 = -80.49 (15) °. Intramolecular O1—H1O1···O2 and O4—H1O4···O3 hydrogen bonds (Table 1) generate two S(5) ring motifs (Fig. 1) (Bernstein *et al.*, 1995). The bond distances and angles in (I) are within normal ranges (Allen *et al.*, 1987).  The absolute configuration of this structure were deduced from an earlier publication (Hueso-Rodríguez *et al.*, 1983). The absolute configuration is R and not S at C10.

The crystal packing of (I) is stabilized by intermolecular O—H···O hydrogen bonds and weak C—H···O interactions (Fig. 2 and Table 1). The molecules are linked into infinite one dimensional chains along the [0 0 1] direction (Fig. 2).

### Experimental

The air-dried roots of *Premna obtusifolia* (4.5 kg) were extracted with CH_2_Cl_2_ (2 *x* 20 *L*) at room temperature. The combined extracts were concentrated under reduced pressure to afford a dark yellow extract (40.5 g) which was subjected to quick column chromatography (QCC) over silica gel using solvents of increasing polarity from n-hexane to EtOAc to afford 12 fractions (F1—F12). Fraction F7 was further purified by QCC using hexane-EtOAc (9.5:0.5), yielding the title compound (212.0 mg). Colourless block-shaped single crystals of the title compound suitable for *x*-ray structure determination were recrystallized from CH_2_Cl_2_/CH_3_OH (1:1, *v*/*v*) after several days.

### Refinement

Hydroxy H atoms attached to O1 and O4 were located from the difference map and isotropically refined. The remaining H atoms were placed in calculated positions with d(O—H) = 0.82 Å and d(C—H) = 0.93 Å for aromatic, 0.98 for CH, 0.97 for CH_2_ and 0.96 Å for CH_3_ atoms. The *U*_iso_ values were constrained to be 1.5*U*_eq_ of the carrier atom for hydroxy and methyl H atoms and 1.2*U*_eq_ for the remaining H atoms. A rotating group model was used for the methyl groups. The highest residual electron density peak is located at 0.67 Å from C12 and the deepest hole is located at 0.47 Å from H1O4. A total of 3037 Friedel pairs were merged before final refinement as there is no large anomalous dispersion for the determination of the absolute configuration.

### Crystal data

**Table:**

C_20_H_26_O_4_	*F*(000) = 712
*M**_r_* = 330.41	*D*_x_ = 1.271 Mg m^−^^3^
Orthorhombic, *P*2_1_2_1_2_1_	Mo *K*α radiation, λ = 0.71073 Å
Hall symbol: P 2ac 2ab	Cell parameters from 3890 reflections
*a* = 9.4946 (1) Å	θ = 2.1–34.0°
*b* = 13.1716 (1) Å	µ = 0.09 mm^−^^1^
*c* = 13.8124 (1) Å	*T* = 100 K
*V* = 1727.37 (3) Å^3^	Block, colourless
*Z* = 4	0.35 × 0.30 × 0.27 mm

### Data collection

**Table:**

Bruker APEXII CCD area-detector diffractometer	3890 independent reflections
Radiation source: sealed tube	3530 reflections with *I* > 2σ(*I*)
graphite	*R*_int_ = 0.031
φ and ω scans	θ_max_ = 34.0°, θ_min_ = 2.1°
Absorption correction: multi-scan (*SADABS*; Bruker, 2005)	*h* = −14→14
*T*_min_ = 0.970, *T*_max_ = 0.977	*k* = −20→19
36584 measured reflections	*l* = −21→21

### Refinement

**Table:**

Refinement on *F*^2^	Primary atom site location: structure-invariant direct methods
Least-squares matrix: full	Secondary atom site location: difference Fourier map
*R*[*F*^2^ > 2σ(*F*^2^)] = 0.039	Hydrogen site location: inferred from neighbouring sites
*wR*(*F*^2^) = 0.114	H atoms treated by a mixture of independent and constrained refinement
*S* = 1.09	*w* = 1/[σ^2^(*F*_o_^2^) + (0.065*P*)^2^ + 0.2832*P*] where *P* = (*F*_o_^2^ + 2*F*_c_^2^)/3
3890 reflections	(Δ/σ)_max_ = 0.001
230 parameters	Δρ_max_ = 0.36 e Å^−^^3^
0 restraints	Δρ_min_ = −0.41 e Å^−^^3^

### Special details

**Table:**

Experimental. The crystal was placed in the cold stream of an Oxford Cryosystems Cobra open-flow nitrogen cryostat (Cosier & Glazer, 1986) operating at 100.0 (1) K.
Geometry. All e.s.d.'s (except the e.s.d. in the dihedral angle between two l.s. planes) are estimated using the full covariance matrix. The cell e.s.d.'s are taken into account individually in the estimation of e.s.d.'s in distances, angles and torsion angles; correlations between e.s.d.'s in cell parameters are only used when they are defined by crystal symmetry. An approximate (isotropic) treatment of cell e.s.d.'s is used for estimating e.s.d.'s involving l.s. planes.
Refinement. Refinement of *F*^2^ against ALL reflections. The weighted *R*-factor *wR* and goodness of fit *S* are based on *F*^2^, conventional *R*-factors *R* are based on *F*, with *F* set to zero for negative *F*^2^. The threshold expression of *F*^2^ > σ(*F*^2^) is used only for calculating *R*-factors(gt) *etc*. and is not relevant to the choice of reflections for refinement. *R*-factors based on *F*^2^ are statistically about twice as large as those based on *F*, and *R*- factors based on ALL data will be even larger.

### Fractional atomic coordinates and isotropic or equivalent isotropic displacement parameters (Å^2^)

**Table:**

	*x*	*y*	*z*	*U*_iso_*/*U*_eq_	
O1	0.26452 (14)	0.14460 (7)	0.64485 (7)	0.0216 (2)	
H1O1	0.269 (3)	0.2000 (18)	0.6119 (16)	0.041 (7)*	
O2	0.25851 (15)	0.33755 (7)	0.66826 (6)	0.0238 (2)	
O3	0.24282 (12)	0.46627 (7)	1.11140 (6)	0.01532 (17)	
H1O3	0.2434	0.5285	1.1112	0.023*	
O4	0.25735 (12)	0.27209 (7)	1.09834 (6)	0.01710 (18)	
H1O4	0.255 (3)	0.3196 (17)	1.1358 (17)	0.036 (6)*	
C1	0.14764 (15)	0.10997 (9)	0.98617 (9)	0.0161 (2)	
H1A	0.1868	0.1047	1.0509	0.019*	
H1B	0.0673	0.1553	0.9895	0.019*	
C2	0.09602 (14)	0.00517 (10)	0.95499 (9)	0.0168 (2)	
H2A	0.0690	−0.0333	1.0119	0.020*	
H2B	0.0132	0.0129	0.9145	0.020*	
C3	0.20820 (14)	−0.05358 (9)	0.89937 (9)	0.0152 (2)	
H3A	0.1749	−0.1219	0.8868	0.018*	
H3B	0.2931	−0.0583	0.9382	0.018*	
C4	0.24219 (15)	−0.00022 (9)	0.80244 (8)	0.01321 (19)	
C5	0.24938 (13)	0.11553 (8)	0.81616 (8)	0.01206 (19)	
C6	0.25713 (15)	0.17857 (9)	0.73871 (8)	0.0149 (2)	
C7	0.25656 (15)	0.28915 (9)	0.74601 (8)	0.0148 (2)	
C8	0.25181 (14)	0.33614 (8)	0.84122 (7)	0.01221 (19)	
C9	0.25195 (13)	0.27427 (8)	0.92386 (7)	0.01059 (18)	
C10	0.26085 (13)	0.15838 (8)	0.91835 (8)	0.01090 (18)	
C11	0.25257 (14)	0.32476 (8)	1.01300 (8)	0.01188 (19)	
C12	0.24742 (13)	0.43145 (8)	1.01841 (7)	0.01168 (19)	
C13	0.24969 (14)	0.49232 (9)	0.93547 (8)	0.01231 (19)	
C14	0.25199 (14)	0.44240 (9)	0.84711 (8)	0.0138 (2)	
H14A	0.2537	0.4803	0.7903	0.017*	
C15	0.24938 (15)	0.60771 (8)	0.94329 (8)	0.0140 (2)	
H15A	0.3063	0.6260	0.9999	0.017*	
C16	0.3149 (2)	0.65916 (11)	0.85549 (11)	0.0284 (3)	
H16A	0.4040	0.6281	0.8413	0.043*	
H16B	0.3287	0.7300	0.8690	0.043*	
H16C	0.2533	0.6519	0.8008	0.043*	
C17	0.09920 (15)	0.64681 (10)	0.96124 (11)	0.0208 (3)	
H17A	0.1012	0.7192	0.9692	0.031*	
H17B	0.0620	0.6159	1.0188	0.031*	
H17C	0.0406	0.6297	0.9070	0.031*	
C18	0.12286 (15)	−0.02886 (11)	0.73086 (10)	0.0202 (3)	
H18A	0.1413	0.0014	0.6689	0.030*	
H18B	0.0345	−0.0044	0.7553	0.030*	
H18C	0.1189	−0.1013	0.7241	0.030*	
C19	0.38326 (14)	−0.04045 (10)	0.76210 (10)	0.0184 (2)	
H19A	0.4574	−0.0258	0.8072	0.028*	
H19B	0.4032	−0.0080	0.7014	0.028*	
H19C	0.3768	−0.1125	0.7526	0.028*	
C20	0.41271 (13)	0.13255 (10)	0.95434 (9)	0.0154 (2)	
H20A	0.4805	0.1608	0.9104	0.023*	
H20B	0.4242	0.0602	0.9570	0.023*	
H20C	0.4267	0.1608	1.0177	0.023*	

### Atomic displacement parameters (Å^2^)

**Table:**

	*U*^11^	*U*^22^	*U*^33^	*U*^12^	*U*^13^	*U*^23^
O1	0.0436 (6)	0.0122 (4)	0.0090 (3)	0.0012 (5)	0.0006 (4)	−0.0013 (3)
O2	0.0489 (6)	0.0129 (4)	0.0097 (3)	0.0008 (5)	0.0007 (4)	0.0023 (3)
O3	0.0266 (4)	0.0104 (4)	0.0090 (3)	−0.0009 (4)	0.0012 (3)	−0.0018 (3)
O4	0.0305 (5)	0.0127 (4)	0.0081 (3)	0.0004 (4)	−0.0006 (4)	0.0015 (3)
C1	0.0209 (5)	0.0107 (5)	0.0166 (5)	−0.0020 (4)	0.0060 (4)	0.0002 (4)
C2	0.0184 (5)	0.0138 (5)	0.0183 (5)	−0.0026 (4)	0.0044 (4)	−0.0001 (4)
C3	0.0205 (5)	0.0093 (5)	0.0160 (5)	−0.0002 (4)	0.0013 (4)	0.0009 (4)
C4	0.0177 (5)	0.0087 (4)	0.0132 (4)	0.0010 (4)	−0.0008 (4)	−0.0007 (3)
C5	0.0156 (5)	0.0094 (4)	0.0112 (4)	0.0009 (5)	−0.0005 (4)	−0.0001 (3)
C6	0.0245 (5)	0.0103 (5)	0.0100 (4)	0.0006 (5)	−0.0002 (4)	−0.0013 (3)
C7	0.0248 (6)	0.0100 (4)	0.0096 (4)	0.0009 (5)	−0.0003 (4)	0.0003 (3)
C8	0.0179 (5)	0.0098 (4)	0.0089 (4)	0.0006 (5)	−0.0001 (4)	0.0001 (3)
C9	0.0141 (4)	0.0084 (4)	0.0093 (4)	0.0002 (4)	0.0002 (4)	0.0002 (3)
C10	0.0140 (4)	0.0088 (4)	0.0100 (4)	0.0003 (4)	−0.0003 (4)	0.0007 (3)
C11	0.0160 (5)	0.0105 (4)	0.0091 (4)	0.0000 (5)	0.0000 (4)	0.0006 (3)
C12	0.0152 (5)	0.0104 (4)	0.0095 (4)	0.0006 (5)	0.0001 (4)	−0.0011 (3)
C13	0.0167 (4)	0.0097 (4)	0.0106 (4)	0.0003 (4)	0.0001 (4)	0.0003 (3)
C14	0.0217 (5)	0.0095 (4)	0.0100 (4)	−0.0001 (5)	0.0002 (4)	0.0001 (3)
C15	0.0223 (5)	0.0077 (4)	0.0121 (4)	0.0001 (5)	0.0018 (4)	0.0000 (3)
C16	0.0519 (10)	0.0117 (5)	0.0215 (6)	0.0005 (6)	0.0146 (6)	0.0016 (5)
C17	0.0232 (6)	0.0134 (5)	0.0259 (6)	0.0024 (5)	−0.0017 (5)	−0.0024 (5)
C18	0.0257 (6)	0.0155 (6)	0.0194 (5)	−0.0024 (5)	−0.0048 (5)	−0.0027 (5)
C19	0.0232 (6)	0.0130 (5)	0.0189 (5)	0.0031 (5)	0.0045 (5)	−0.0007 (4)
C20	0.0164 (5)	0.0130 (5)	0.0168 (5)	0.0017 (4)	−0.0034 (4)	−0.0003 (4)

### Geometric parameters (Å, °)

**Table:**

O1—C6	1.3733 (14)	C9—C11	1.3993 (15)
O1—H1O1	0.86 (2)	C9—C10	1.5307 (16)
O2—C7	1.2490 (14)	C10—C20	1.5626 (17)
O3—C12	1.3645 (13)	C11—C12	1.4081 (15)
O3—H1O3	0.8200	C12—C13	1.3984 (15)
O4—C11	1.3685 (13)	C13—C14	1.3864 (15)
O4—H1O4	0.81 (2)	C13—C15	1.5237 (16)
C1—C2	1.5268 (18)	C14—H14A	0.9300
C1—C10	1.5619 (17)	C15—C16	1.5222 (18)
C1—H1A	0.9700	C15—C17	1.5362 (19)
C1—H1B	0.9700	C15—H15A	0.9800
C2—C3	1.5242 (18)	C16—H16A	0.9600
C2—H2A	0.9700	C16—H16B	0.9600
C2—H2B	0.9700	C16—H16C	0.9600
C3—C4	1.5462 (17)	C17—H17A	0.9600
C3—H3A	0.9700	C17—H17B	0.9600
C3—H3B	0.9700	C17—H17C	0.9600
C4—C5	1.5378 (16)	C18—H18A	0.9600
C4—C19	1.5444 (19)	C18—H18B	0.9600
C4—C18	1.5504 (18)	C18—H18C	0.9600
C5—C6	1.3562 (16)	C19—H19A	0.9600
C5—C10	1.5240 (15)	C19—H19B	0.9600
C6—C7	1.4600 (16)	C19—H19C	0.9600
C7—C8	1.4542 (15)	C20—H20A	0.9600
C8—C14	1.4020 (15)	C20—H20B	0.9600
C8—C9	1.4025 (14)	C20—H20C	0.9600
			
C6—O1—H1O1	103.0 (15)	O4—C11—C9	121.14 (10)
C12—O3—H1O3	109.5	O4—C11—C12	117.47 (9)
C11—O4—H1O4	99.1 (16)	C9—C11—C12	121.39 (10)
C2—C1—C10	114.88 (10)	O3—C12—C13	125.37 (10)
C2—C1—H1A	108.5	O3—C12—C11	112.74 (9)
C10—C1—H1A	108.5	C13—C12—C11	121.88 (10)
C2—C1—H1B	108.5	C14—C13—C12	116.71 (10)
C10—C1—H1B	108.5	C14—C13—C15	122.37 (10)
H1A—C1—H1B	107.5	C12—C13—C15	120.91 (10)
C3—C2—C1	112.14 (11)	C13—C14—C8	121.63 (10)
C3—C2—H2A	109.2	C13—C14—H14A	119.2
C1—C2—H2A	109.2	C8—C14—H14A	119.2
C3—C2—H2B	109.2	C16—C15—C13	112.76 (10)
C1—C2—H2B	109.2	C16—C15—C17	111.02 (12)
H2A—C2—H2B	107.9	C13—C15—C17	110.34 (11)
C2—C3—C4	110.58 (10)	C16—C15—H15A	107.5
C2—C3—H3A	109.5	C13—C15—H15A	107.5
C4—C3—H3A	109.5	C17—C15—H15A	107.5
C2—C3—H3B	109.5	C15—C16—H16A	109.5
C4—C3—H3B	109.5	C15—C16—H16B	109.5
H3A—C3—H3B	108.1	H16A—C16—H16B	109.5
C5—C4—C19	110.25 (11)	C15—C16—H16C	109.5
C5—C4—C3	110.69 (9)	H16A—C16—H16C	109.5
C19—C4—C3	109.72 (10)	H16B—C16—H16C	109.5
C5—C4—C18	110.63 (10)	C15—C17—H17A	109.5
C19—C4—C18	108.68 (10)	C15—C17—H17B	109.5
C3—C4—C18	106.80 (10)	H17A—C17—H17B	109.5
C6—C5—C10	119.99 (10)	C15—C17—H17C	109.5
C6—C5—C4	120.81 (10)	H17A—C17—H17C	109.5
C10—C5—C4	118.97 (9)	H17B—C17—H17C	109.5
C5—C6—O1	123.23 (11)	C4—C18—H18A	109.5
C5—C6—C7	123.79 (10)	C4—C18—H18B	109.5
O1—C6—C7	112.98 (10)	H18A—C18—H18B	109.5
O2—C7—C8	124.11 (10)	C4—C18—H18C	109.5
O2—C7—C6	116.73 (10)	H18A—C18—H18C	109.5
C8—C7—C6	119.15 (10)	H18B—C18—H18C	109.5
C14—C8—C9	122.20 (10)	C4—C19—H19A	109.5
C14—C8—C7	118.51 (10)	C4—C19—H19B	109.5
C9—C8—C7	119.25 (10)	H19A—C19—H19B	109.5
C11—C9—C8	116.10 (10)	C4—C19—H19C	109.5
C11—C9—C10	121.16 (9)	H19A—C19—H19C	109.5
C8—C9—C10	122.62 (9)	H19B—C19—H19C	109.5
C5—C10—C9	114.30 (9)	C10—C20—H20A	109.5
C5—C10—C1	110.80 (10)	C10—C20—H20B	109.5
C9—C10—C1	109.83 (9)	H20A—C20—H20B	109.5
C5—C10—C20	106.26 (10)	C10—C20—H20C	109.5
C9—C10—C20	104.61 (10)	H20A—C20—H20C	109.5
C1—C10—C20	110.81 (9)	H20B—C20—H20C	109.5
			
C10—C1—C2—C3	29.06 (15)	C6—C5—C10—C20	−103.51 (13)
C1—C2—C3—C4	−65.02 (14)	C4—C5—C10—C20	71.15 (14)
C2—C3—C4—C5	41.24 (14)	C11—C9—C10—C5	176.26 (11)
C2—C3—C4—C19	163.14 (10)	C8—C9—C10—C5	−7.91 (17)
C2—C3—C4—C18	−79.24 (12)	C11—C9—C10—C1	51.02 (15)
C19—C4—C5—C6	68.23 (16)	C8—C9—C10—C1	−133.15 (12)
C3—C4—C5—C6	−170.18 (12)	C11—C9—C10—C20	−67.95 (14)
C18—C4—C5—C6	−52.01 (17)	C8—C9—C10—C20	107.89 (13)
C19—C4—C5—C10	−106.38 (12)	C2—C1—C10—C5	25.02 (15)
C3—C4—C5—C10	15.20 (16)	C2—C1—C10—C9	152.24 (11)
C18—C4—C5—C10	133.38 (11)	C2—C1—C10—C20	−92.68 (13)
C10—C5—C6—O1	171.98 (12)	C8—C9—C11—O4	−177.87 (12)
C4—C5—C6—O1	−2.6 (2)	C10—C9—C11—O4	−1.77 (19)
C10—C5—C6—C7	−8.9 (2)	C8—C9—C11—C12	2.54 (19)
C4—C5—C6—C7	176.56 (13)	C10—C9—C11—C12	178.63 (11)
C5—C6—C7—O2	−177.17 (14)	O4—C11—C12—O3	−2.26 (19)
O1—C6—C7—O2	2.0 (2)	C9—C11—C12—O3	177.35 (11)
C5—C6—C7—C8	2.0 (2)	O4—C11—C12—C13	176.62 (11)
O1—C6—C7—C8	−178.82 (12)	C9—C11—C12—C13	−3.8 (2)
O2—C7—C8—C14	−1.2 (2)	O3—C12—C13—C14	−178.94 (13)
C6—C7—C8—C14	179.71 (13)	C11—C12—C13—C14	2.33 (19)
O2—C7—C8—C9	−179.12 (14)	O3—C12—C13—C15	1.0 (2)
C6—C7—C8—C9	1.8 (2)	C11—C12—C13—C15	−177.76 (12)
C14—C8—C9—C11	−0.13 (19)	C12—C13—C14—C8	0.1 (2)
C7—C8—C9—C11	177.69 (12)	C15—C13—C14—C8	−179.81 (13)
C14—C8—C9—C10	−176.16 (12)	C9—C8—C14—C13	−1.2 (2)
C7—C8—C9—C10	1.66 (19)	C7—C8—C14—C13	−179.04 (12)
C6—C5—C10—C9	11.32 (18)	C14—C13—C15—C16	−25.4 (2)
C4—C5—C10—C9	−174.02 (10)	C12—C13—C15—C16	154.74 (13)
C6—C5—C10—C1	136.05 (13)	C14—C13—C15—C17	99.41 (15)
C4—C5—C10—C1	−49.30 (15)	C12—C13—C15—C17	−80.49 (15)

### Hydrogen-bond geometry (Å, °)

**Table:**

*D*—H···*A*	*D*—H	H···*A*	*D*···*A*	*D*—H···*A*
O1—H1O1···O2	0.86 (2)	1.97 (2)	2.5626 (13)	124.5 (19)
O3—H1O3···O2^i^	0.82	1.93	2.7008 (13)	156
O4—H1O4···O3	0.81 (2)	1.96 (2)	2.5677 (13)	131 (2)
C14—H14A···O3^ii^	0.93	2.57	3.4712 (14)	163
C15—H15A···O2^i^	0.98	2.45	3.1908 (14)	132
C18—H18A···O1	0.96	2.24	2.9053 (18)	125
C19—H19B···O1	0.96	2.53	3.1360 (17)	121
C20—H20C···O4	0.96	2.44	3.0838 (16)	124

Symmetry codes:  (i) −*x*+1/2, −*y*+1, *z*+1/2; (ii) −*x*+1/2, −*y*+1, *z*−1/2.
